# Improving QRISK Assessment in Patients Admitted with Severe Mental Illness

**DOI:** 10.1192/bjo.2026.11402

**Published:** 2026-06-30

**Authors:** Upeshala Jayawardena, Vasudevan Krishnan

**Affiliations:** 1 University Hospitals of North Midlands NHS Trust, Stoke-on-Trent, United Kingdom; 2 St Georges Hospital, Stafford, United Kingdom

## Abstract

**Aims:**

Severe mental illness (SMI) is associated with significant cardiovascular disease due to a combination of lifestyle factors, metabolic effects of antipsychotic medications and reduced access to preventive healthcare. This quality improvement project (QIP) wasconducted to enhance cardiovascular risk assessment in patients with SMI by improving the efficiency and consistency of QRISK score documentation.

**Methods:**

This study was conducted over two cycles involving patients admitted with SMI to a general psychiatric unit. Cycle 1 covered a five-month period (December 2024–May 2025), and Cycle 2 a four-month period (July 2025–October 2025). Cardiovascular risk was assessed using QRISK®3 (2018) tool, with eligible patients aged 25–84 years included. Thirty patients were analysed in Cycle 1 and 27 in Cycle 2. Data were retrospectively collected from patient’s electronic records and physical health parameters. Following baseline assessment, a standardised algorithm was implemented to improve consistency of QRISK calculation. Cycle 2 reassessed outcomes to evaluate the impact of this intervention.

**Results:**

In Cycle 1, 30 patients with severe mental illness (SMI) were included, of whom 70% were male. In contrast, Cycle 2 comprised 27 patients, with females accounting for 59% of admissions. The age range was 26–75 years in Cycle 1 and 32–63 years in Cycle 2. Schizophrenia was the most common diagnosis in both cycles (36% in Cycle 1 and 33% in Cycle 2), followed by bipolar affective disorder (20% and 18%, respectively). Aripiprazole was the most frequently prescribed antipsychotic in both cycles (37% in Cycle 1 and 25% in Cycle 2).

During Cycle 1, QRISK scores were not documented for 15 patients (50%). Following implementation of the clinical algorithm, this improved in Cycle 2, with only 5 patients (18.5%) lacking a recorded QRISK score. In Cycle 1, a total of 8 patients were identified as having QRISK scores >10%, of whom 2 had not been documented. In Cycle 2, 6 patients had QRISK scores >10%, with no patients missed, following intervention.

**Conclusion:**

This QIP highlighted the importance of routine QRISK assessment in patients admitted with SMI, who are at increased risk of cardiovascular disease. Implementation of a structured clinical algorithm significantly reduced missed QRISK calculations, improving identification of high-risk individuals. Continued staff education and strengthened collaboration between mental health and primary care services may further enhance follow-up and long-term cardiovascular risk management in this population.

